# Acupuncture plus Low-Frequency Electrical Stimulation (Acu-LFES) Attenuates Diabetic Myopathy by Enhancing Muscle Regeneration

**DOI:** 10.1371/journal.pone.0134511

**Published:** 2015-07-31

**Authors:** Zhen Su, Alayna Robinson, Li Hu, Janet D. Klein, Faten Hassounah, Min Li, Haidong Wang, Hui Cai, Xiaonan H. Wang

**Affiliations:** 1 Department of Nephrology, The First Affiliated Hospital of Wenzhou Medical University, Wenzhou, Zhejiang, China; 2 Acumox and Tuina Research Section, College of Acumox and Tuina, Shanghai University of Traditional Chinese Medicine, Shanghai, China; 3 Renal Division, Department of Medicine, Emory University, Atlanta, GA, United States of America; University of Louisville School of Medicine, UNITED STATES

## Abstract

Mortality and morbidity are increased in patients with muscle atrophy resulting from catabolic diseases such as diabetes. At present there is no pharmacological treatment that successfully reverses muscle wasting from catabolic conditions. We hypothesized that acupuncture plus low frequency electric stimulation (Acu-LFES) would mimic the impact of exercise and prevent diabetes-induced muscle loss. Streptozotocin (STZ) was used to induce diabetes in mice. The mice were then treated with Acu-LFES for 15 minutes daily for 14 days. Acupuncture points were selected according to the WHO Standard Acupuncture Nomenclature guide. The needles were connected to an SDZ-II electronic acupuncture device delivering pulses at 20Hz and 1mA. Acu-LFES prevented soleus and EDL muscle weight loss and increased hind-limb muscle grip function in diabetic mice. Muscle regeneration capacity was significantly increased by Acu-LFES. The expression of Pax7, MyoD, myogenin and embryo myosin heavy chain (eMyHC) was significantly decreased in diabetic muscle *vs*. control muscle. The suppressed levels in diabetic muscle were reversed by Acu-LFES. The IGF-1 signaling pathway was also upregulated by Acu-LFES. Phosphorylation of Akt, mTOR and p70S6K were downregulated by diabetes leading to a decline in muscle mass, however, Acu-LFES countered the diabetes-induced decline. In addition, microRNA-1 and -206 were increased by Acu-LFES after 24 days of treatment. We conclude that Acu-LFES is effective in counteracting diabetes-induced skeletal muscle atrophy by increasing IGF-1 and its stimulation of muscle regeneration.

## Introduction

Muscle wasting is an independent index for mortality and morbidity in catabolic conditions [1, 2], such as diabetes, chronic kidney disease, heart failure, cancer, sepsis, burn injury, AIDS and denervation [3–6]. Muscle loss is mainly reflective of increased myofibrillar protein breakdown [7]. Loss of muscle protein results in muscle atrophy and weakness. These have significant clinical consequences, up to and including decreased life expectancy. Therefore, effective therapeutic strategies to treat muscle wasting induced by catabolic diseases are needed.

The prevalence of diabetes worldwide is increasing [8]. Diabetes affects more than ten million Americans. The routine injection of insulin before meals has substantially improved the lives of people who suffer from diabetes by controlling their hyperglycemia, but this does not address the other aspects of diabetes. There is an overall failure of this treatment to eliminate the development of long-term complications that result from insulin resistance. We have evidence that insulin resistance and/or insulin deficiency will cause muscle weakness in diabetes [4, 9, 10]. This clinical condition is termed diabetic myopathy, which is a reduction in skeletal muscle size and strength [11]. Diabetic myopathy is a significant, but often overlooked, complication that contributes an overall worsening of the diabetic condition. Maintenance of healthy muscle capacity is of vital importance for our physical and metabolic well-being. In addition to locomotion, skeletal muscle constitutes about 40% of our body mass and is a major metabolic organ system. A key challenge in diabetes is to maintain muscle mass which requires muscle regeneration.

Muscle regeneration is a multistep process [12]. The first step is to activate myogenic stem cells, also known as satellite cells. Pax7 is a transcription factor that activates satellite cells leading to initial myogenesis [13]. After initiation, the muscle cells can either proliferate or return to quiescence. MyoD is a marker of myogenic commitment to muscle proliferation and differentiation. Following differentiation, the cells need to fuse either with new or previously existing fibers during the process of differentiation. Myogenin is required for the fusion of myogenic precursor cells. At this point the muscle has regenerated to a mature form that is capable of contraction. Myosin comprises a family of ATP-dependent motor proteins and is best known for their role in muscle contraction. They are responsible for actin-based motility. Myosin heavy chain protein is a marker indicating mature muscle. Together, these markers give a comprehensive picture of the muscle regeneration status in either healthy or diabetic animals.

Previously we and others discovered that exercise has the ability to prevent a decrease of skeletal muscle mass [5]. Unfortunately, patients with severe diseases are frequently unable to consistently exercise. Although substantial efforts have been made to understand and treat muscle wasting, pharmacologic treatments for muscle wasting have had limited success. Acupuncture is a branch of traditional Chinese medicine that is widely applied to treat various diseases. Acupuncture plus Low-frequency electrical stimulation (Acu-LFES) is an acupuncture technique that replicates the benefits of exercise through stimulation of muscle contraction. Our previous studies found that Acu-LFES is a non-pharmacologic approach that can prevent muscle loss induced by chronic kidney disease [14].

In this study, we investigated the impact of Acu-LFES on diabetic myopathy. We believe that regardless of the underlying cause or catabolic condition, there are common mechanisms that are part of the muscle wasting process. One such commonality is a decrease in insulin-like growth factor 1 (IGF-1) signaling that leads to muscle wasting. We hypothesized that Acu-LFES would upregulate the IGF-1 signaling pathway, resulting in suppressed diabetes-induced muscle loss. We report here that Acu-LFES improves muscle health by influencing muscle regeneration in diabetes. The positive effects of Acu-LFES could provide an additional therapeutic option for treatment of diabetic myopathy.

## Materials and Methods

### Animals and diabetic model

Mice (C57BL/6J, 8–10 weeks, male) were housed in the animal care facility in 12-h light, 12-h dark cycles and fed ad libitum with normal chow. The experiments were approved by the institutional animal care and use committee (IACUC) of Emory University. Streptozotocin (STZ) induction of diabetes and Acu-LFES were described in detail in the Complete Research Description section of approved IACUC Protocol number: DAR-2002853. Mice were randomly assigned into four groups: control, Acu-LFES, diabetes and diabetes/Acu-LFES (n = 12/group). Diabetes was induced by STZ injection [10, 15]. STZ-Na-Citrate solution was freshly prepared immediately prior to injection. Mice were injected with STZ in citrate buffer (1.47 g of Na Citrate in 50 ml ddH_2_O, adjust buffer to pH 4.5 with monohydrate Na Citrate) at a dose of 150 mg/kg mouse. The final concentration of STZ in the Na-Citrate buffer was 22.5 mg/ml. Each mouse received injections on two consecutive days. Blood glucose was measured by Accu-Chek Aviva Plus Test Strips (Roche Diagnostics, Indianapolis, IN). At the end of the experiment, mice were euthanatized by cervical dislocation. The gastrocnemius, soleus and extensor digitorum longus (EDL) muscles were removed, muscle weights were measured and then muscles were immersed in liquid nitrogen and stored at -80°C for further experiments. The muscles were analyzed for mRNA and protein in a combined sample because this more accurately reflects the comprehensive muscle response to the acupuncture treatment.

### Muscle grip function

Grip strength was measured using a mouse grip strength meter with dual computerized sensors to detect and record the grip force (Columbus Instruments, Columbus, OH). Mice were allowed to grip a grid connected to a force transducer and gently pulled by the tail for 5 seconds. The computerized sensors determine what force was needed to counter-balance the grip of the mice. Mice were tested before the STZ injection and Acu-LFES treatment and 14 days after Acu-LFES just before harvesting the muscle. The grip strength of each mouse was tested 5 times on each testing occasion, with 10 minutes rest between each test. The average of the 5 determinations was reported.

### Acu-LFES treatment

The mice were kept in specially designed restraint so that they would remain in a recumbent position during Acu-LFES treatment. Acupuncture points were selected according to the WHO Standard Acupuncture guidelines [16]. The positive point (Yang Ling Quan, GB34) is in the hollow of the exterior-inferior of the caput fibulae about 6 mm deep. This position is close to the superficial fibular nerve and deep fibular nerve. The negative point (Zu San Li, ST36) is 5 mm beneath the capitulum fibulae and located laterally and posterior to the knee-joint about 7 mm deep and close to fibular nerve. The needles were connected to an SDZ-II Electronic acupuncture instrument using consistent pulse, electric frequency 20Hz, electric current 1mA. The Acu-LFES was administered for 15 minutes every day for 14 days. Disposable sterile needles with a diameter of 0.25 mm (Shen Li Medical & Health Material Co., Ltd., Wujiang, China) were used.

### Western blot and antibodies

Hind limb muscles were homogenized in RIPA Buffer. Proteins were subjected to Western blot analysis using previously published methods [9]. Primary antibodies included: Akt/p-Akt (Ser473), FoxO1/p-FoxO1 (Thr32), mTOR/p-mTOR (Ser2448), 70S6K/p-p70S6K (Thr389) were purchased from Cell Signaling (Danvers, MA). MyoD, Pax7, Myogenin, eMyHC are from DSHB product (University of Iowa, lowa City, IA). GAPDH is from Millipore (Burlington, MA). Antibodies were used at a 1:1000 dilution except where indicated. Protein bands were scanned and quantified using the Li-cor Odyssey infrared scanning system (Li-COR Biosciences, Lincoln, NE).

### Muscle Immunohistology

The method for immunohistology of muscle cross sections has been previously described [10]. Muscles were embedded in TBS Tissue Freezing Media (Fisher, Pittsburgh, PA) in isopentane cooled in dry ice. Muscle cross sectional slices (10 mm) were mounted on gelatin-coated slides were fixed in 4% paraformaldehyde for 10 min. Tissue was permeabilized in 0.05% Triton X-100 (in PBS) for 10 min, and quench-fixed in 50 mM NH_4_Cl for another 10 min. Samples were blocked with 5% bovine serum albumin for 1 h, followed by incubation overnight with polyclonal anti-laminin antibody (Sigma-Aldrich). Sections were further incubated for 60 min with FITC donkey anti-rabbit IgG (Jackson Immuno Research Lab, West Grove, PA). Nuclei were stained with DAPI. Images were visualized with an Olympus 1X51 inverted fluorescence microscope and captured by DP73-1-51-17MP color camera. The muscle fiber cross sectional area for each muscle was an average of at least 500 individual myofibers, measured using the cellSens Dimension 1.9 Software (Olympus, Melville, NY). The size of the myofibers reported reflects an average from six mice per group.

### Quantitative measurement of mouse IGF-1 in muscle lysate

IGF-1 Mouse Enzyme-Linked Immunosorbent Assay (ELISA) Kit (ab100695) was purchased from Abcam (Cambridge, MA) and used according to manufacturer’s instructions.

### Reverse transcription and quantitative polymerase chain reaction (q-PCR) for mRNA and microRNA

Total RNA was extracted using Tri-Reagent (Molecular Research Inc., Cincinnati, OH). RNA was subjected to reverse transcription and qPCR using previously published methods [17]. For microRNA, the miRCURY LNA Universal cDNA Synthesis kit (Exiqon Inc., Woburn, MA) was used for reverse transcription of microRNA. The primers were custom designed by Exiqon. The miRCURY LNA microRNA PCR SYBR Green master mix (Exiqon INC) was used for qPCR with the following cycle parameters: 95°C for 10 minutes and 45 cycles at 95°C for 10 seconds and 60°C for 60 seconds. Expression of individual miRNA was normalized to the mouse U6 gene and calculated as the difference between the threshold values of the two genes (ΔΔcq).

### Conventional Reverse transcription PCR (RT-PCR) for mRNA

Total RNA from mouse muscle was isolated using Tri-Reagent. Reverse transcription was performed using the M-MLV reverse transcriptase (Life Technologies, Grand Island, NY) and 2 μg denatured RNA according to the manufacturer's instructions. Primers for specific genes were designed to cross intron-exon boundaries and used to generate amplicons in their linear ranges. For each sample, 18S rRNA was used as an internal control. The following primers were used for RT-PCR: MyoD (M84918), forward 5’- GCC CGC GCT CCA ACT GCT CTG AT -3’, reverse 5’- CCT ACG GTG GTG CGC CCT CTG C -3’ (amplicon 397 nt); Pax-7 (NM_01039), forward 5’- TGG AAG TGT CCA CCC CTC TTG GC -3’, reverse 5’- ATC CAG ACG GTT CCC TTT GTC GCC -3’ (amplicon 510 nt); eMyHC (M11154), forward 5’- GAA GAA GAA CCT GGA GCA GAC G -3’, reverse 5’- AGC CTG CCT CTT GTA GGA CTT G-3’ (amplicon 301 nt) and Myogenin (NM_031189), forward 5’- TGC ACT CCC TTA CGT CCA TCG T-3’, reverse 5’- AGG TCA GGG CAC TCA TGT CTC T-3’ (amplicon 454 nt).

### Statistical analysis

Data are presented as mean ± se. To identify significant differences between two groups, comparisons were made using a Student’s t-test. For a comparison of more than two groups, ANOVA was performed with a post hoc analysis by the Student-Newman-Keuls test. Differences with P values < 0.05 were considered significant.

## Results

### Acu-LFES prevents diabetes-induced muscle wasting and improves muscle function

In diabetic mice, blood glucose values were three times higher than those of control mice (*P* < 0.01). The weights of soleus (slow-contracting dark type I fiber) and EDL (fast-twitch white type II fiber) muscles in mice with diabetes were significantly less than those of control mice, but those of non-diabetic mice treated with Acu-LFES were not significantly different from those of control mice (Table 1). However, the muscle weights were significantly higher in diabetic mice with Acu-LFES than in diabetic mice without Acu-LFES. The muscle function of the mice, as measured by the grip strength meter, is shown in Table 2. There was no significant difference in muscle function between non-diabetic mice with and those without Acu-LFES. Diabetic mice had lower grip function than did control mice, but diabetic mice treated with Acu-LFES had higher muscle grip capacity than did diabetic mice without Acu-LFES (diabetes: 3.8 ± 0.9 KGF^−2^; diabetes/Acu-LFES: 5.2 ± 1.0 KGF^−2^; *P* < 0.05).

**Table 1:** Muscle and body Weights.

	control	Acu-LFES	Diabetes	Diabetes/Acu-LFES
Body weight (g)	24.9 ± 2.9	24.1 ± 2.6	21.3 ± 1.9	23.2 ± 2.6
Soleus (mg)	11.1 ± 2.0	11.8 ± 1.7	8.3 ± 1.5*	10.0 ±1.7^#^
EDL (mg)	10.8 ± 1.3	11.0 ± 1.6	8.4 ± 2.0*	9.9 ± 1.5^#^
gastrocnemius (mg)	140.4 ± 9.3	145.0 ± 11.9	118.4 ± 12.8*	138.9 ± 11.3^#^
soleus/body (x100)	44.2 ± 3.9	48.9 ± 4.1	39.0 ± 3.2*	43.8 ± 2.8^#^
EDL/body (x100)	43.4 ± 2.4	45.6 ± 1.2	39.4 ± 5.2*	42.7 ± 4.7^#^
Gastrocnemius/body (x100)	563.9 ± 8.2	601.7 ± 7.6	553.9 ± 9.7	594.8 ± 8.3
Blood glucose (mg %)	100.3 ± 6.1	102.2 ± 5.3	303.2 ± 5.3*	289.3 ± 16.7*

Data are presented as mean ± s.e.;

P < 0.05 is significant (*vs. control, ^#^ *vs*. Diabetes), n = 12/group.

**Table 2:** Muscle function was increased by LFES.

	Before Acu-LFES (KGF^-2^)	After Acu-LFES (KGF^-2^)
Control	4.9 ± 0.2	5.1 ± 0.6
Acu-LFES	4.7 ± 0.6	6.6 ± 0.8
Diabetes	5.0 ± 0.4	3.8 ± 0.9*
Diabetes/Acu-LFES	4.8 ± 0.4	5.2 ± 1.0^#^

P < 0.05 is significant (*vs. control, ^#^vs. diabetes), n = 12/group.

KGF^-2^, kilogram-force/100; Data are presented as mean ± s.e.;

### Acu-LFES prevents diabetes-induced muscle fiber cross-sectional area decrease

**Fig 1:**
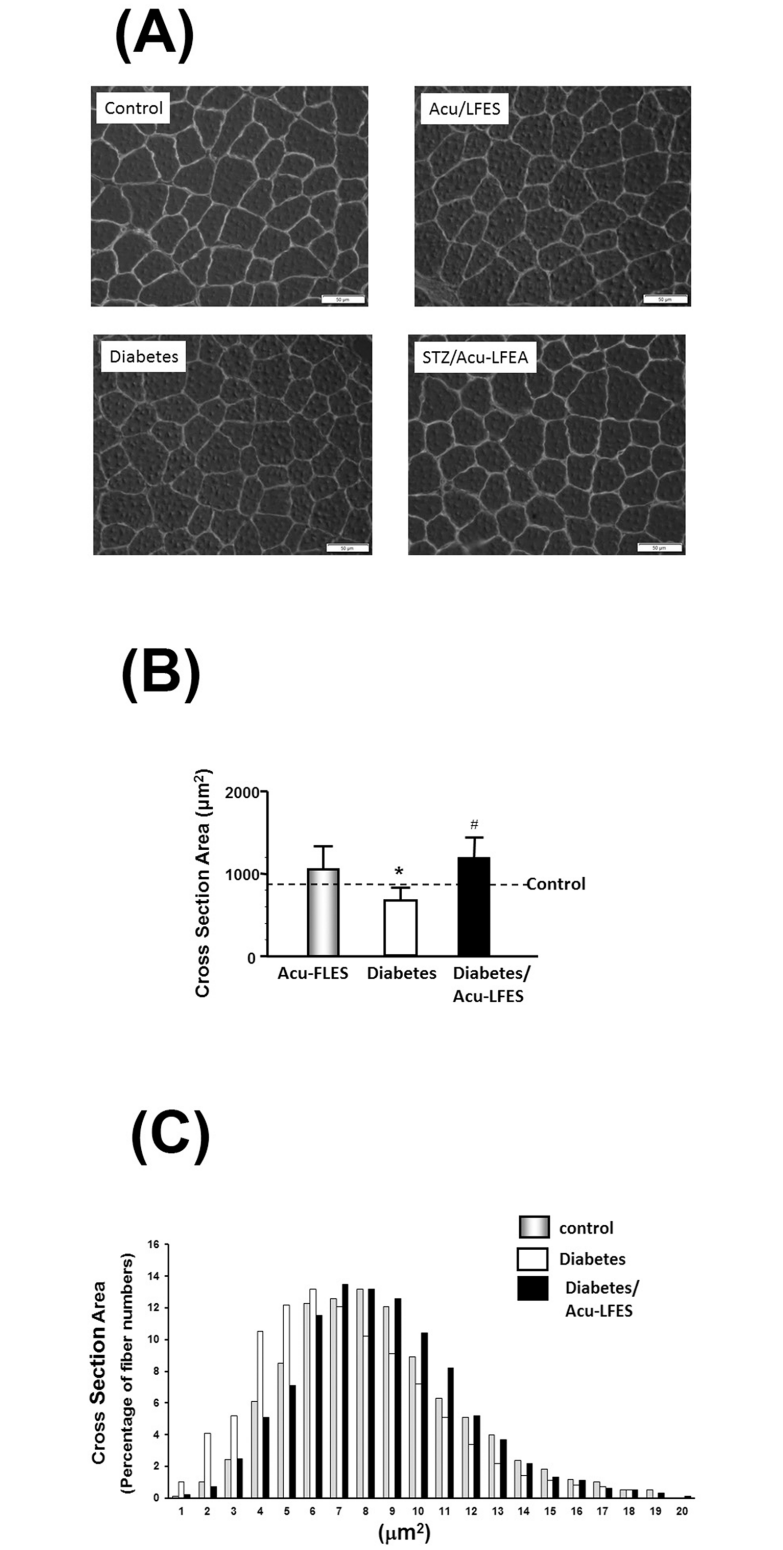
Acu-LFES prevents diabetes-induced muscle fiber cross-sectional area (MCS) decrease. A representative cross-sectional area of EDL muscle from normal control (control), Acu-LFES treated normal controls (Acu-LFES), diabetes or diabetes/Acu-LFES mice are showed (A). The cryosections of EDL muscle were immunostained with anti-laminin antibody. The First bar graph (B) shows the average size of myofibers determined from six mice x 8 sections/mouse/group (Bars: mean ± s.e.; n = 9/group; * = p<0.05 vs. control and # = p<0.05 vs. diabetes). The frequency distribution of fiber cross-sectional area in control (grey bar), diabetes (open bar) and diabetes/Acu-LFES (black bar) mice is presented as percent fibers/size of fibers (C).

Muscle fiber cross-sectional area was determined in frozen sections of EDL muscles using an anti-laminin antibody. The size of muscle fibers was significantly smaller in diabetic mice without Acu-LFES than in diabetic mice with Acu-LFES. Fiber area frequency distribution revealed a clear increase in the percentage of small fibers (a leftward shift) in diabetic mice (Fig 1). Acu-LFES in diabetic mice suppressed the leftward shift in the fiber size distribution.

### Acu-LFES improves muscle regeneration in normal and diabetic mice

**Fig 2:**
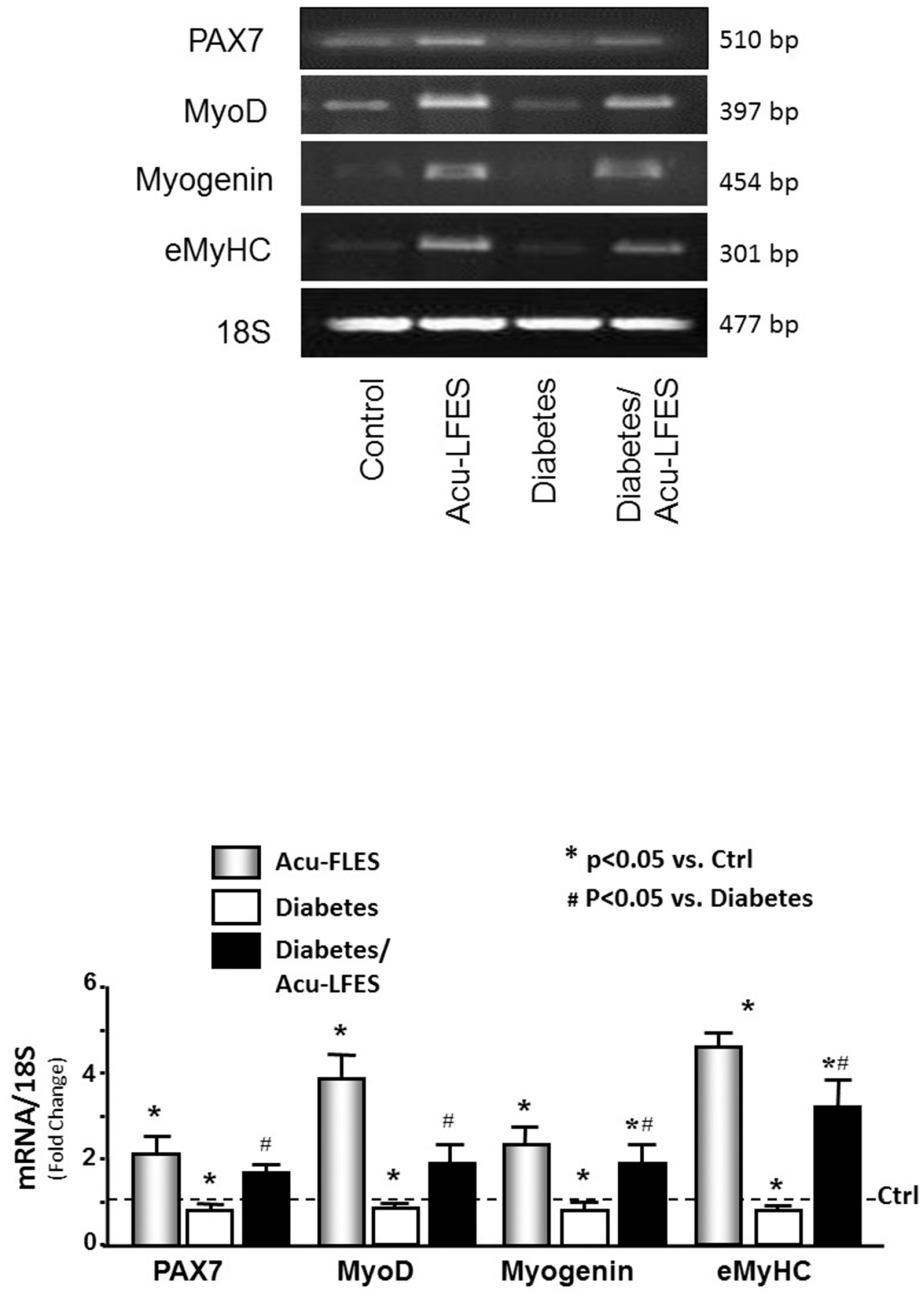
Acu-LFES increases muscle regeneration of mRNA markers. Pax7, myoD, myogenin and eMyHC were measured by conventional RT-PCR in combined gastrocnemius and EDL muscle lysates from control, Acu-LFES, diabetes or diabetes/Acu-LFES mice. The bar graph compares the densities of mRNA bands in each group expressed as a fold-change from levels in control mice which is represented by a line at 1-fold. All band densities were normalized to the density of the 18S rRNA band (Bars: mean ± s.e.; n = 12/group; * = p<0.05 vs. control and # = p<0.05 vs. diabetes).

**Fig 3:**
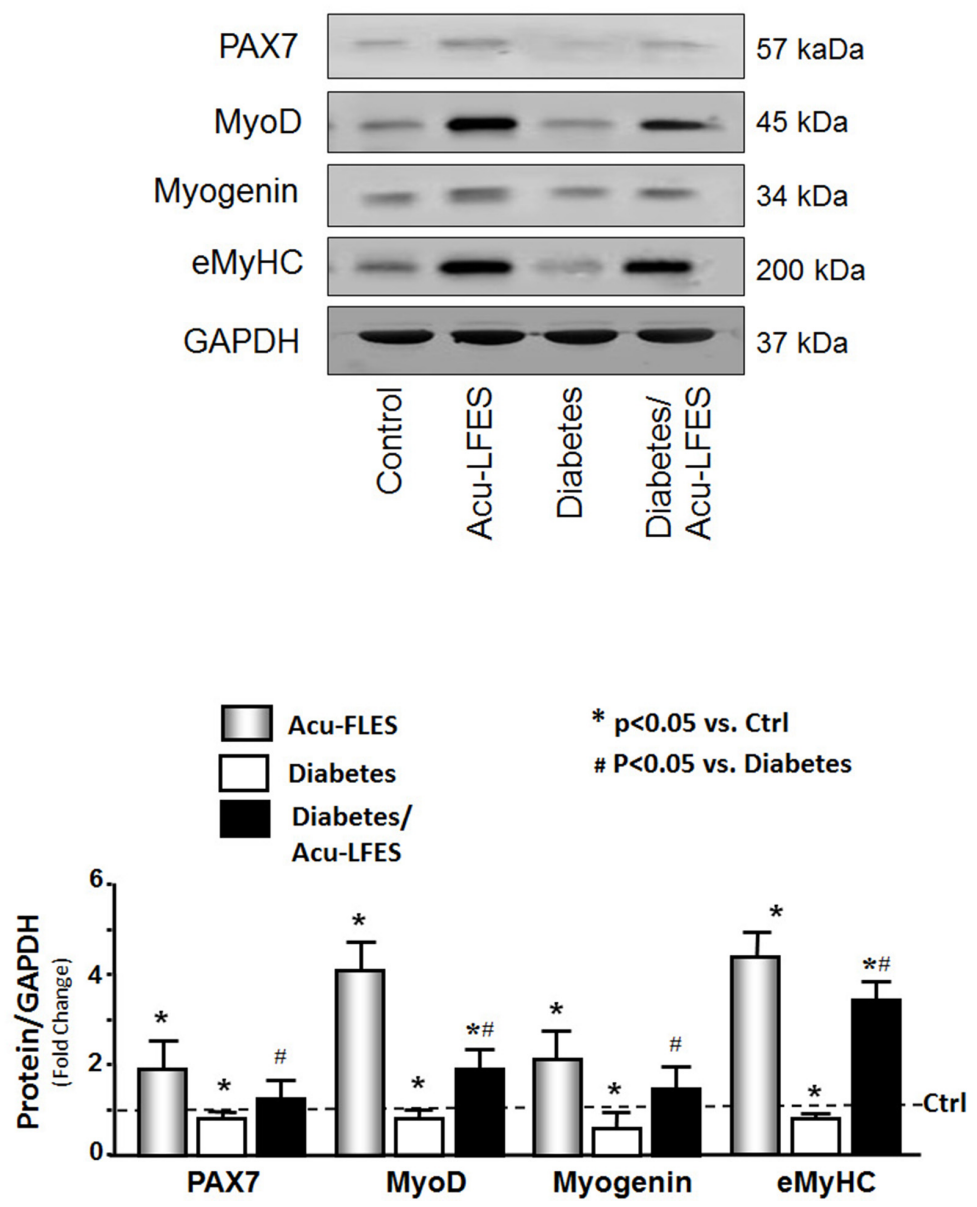
Acu-LFES counteracts diabetes-induced decrease of muscle regeneration proteins. Muscle proteins lysates were prepared from combined gastrocnemius and EDL muscles from control, Acu-LFES, diabetes or diabetes/Acu-LFES mice. Muscle regeneration related proteins (Pax7, myoD, myogenin, eMyHC) and GAPDH were measured by western blotting. The bar graph compares the protein band densities in each treatment group expressed as a fold-change from levels in control mice (represented by a line at 1-fold). All band densities were normalized to the density of GAPDH (Bars: mean ± s.e.; n = 12/group; * = p<0.05 vs. control and # = p<0.05 vs. diabetes).

To study how Acu-LFES prevents muscle wasting in diabetic mice, we first measured the mRNA of myogenesis markers. In the muscles of diabetic mice, the mRNA expressions of *PAX7* (transcription factor to initial myogenesis), *MYOD* (proliferation marker), myogenin (differentiation marker), and e*MyHC* (embryo myosin heavy chain differentiation and fusion marker) were significantly lower than those of control mice (Fig 2). Acu-LFES reversed the diabetes-induced suppression of myogenesis mRNA. The protein levels of muscle regeneration markers also provide evidence that Acu-LFES stimulates muscle regeneration capacity in both healthy and diabetic mice (Fig 3). All myogenesis markers tested were increased by Acu-LFES in healthy controls: by 1.9-fold for PAX7, 4.1-fold for MYOD, 2.1-fold for myogenin, and 4.3-fold for eMyHC. The PAX7, MYOD, and eMyHC markers were significantly decreased in muscles of diabetic mice; Acu-LFES reversed these decreases. Myogenin protein was also decreased in the muscles of diabetic mice, but the change was not statistically significant. However, among diabetic mice, myogenin was significantly higher in those treated with Acu-LFES than in those not treated with Acu-LFES. These results show that Acu-LFES prevents diabetes-induced muscle wasting by stimulating muscle regeneration.

**Fig 4:**
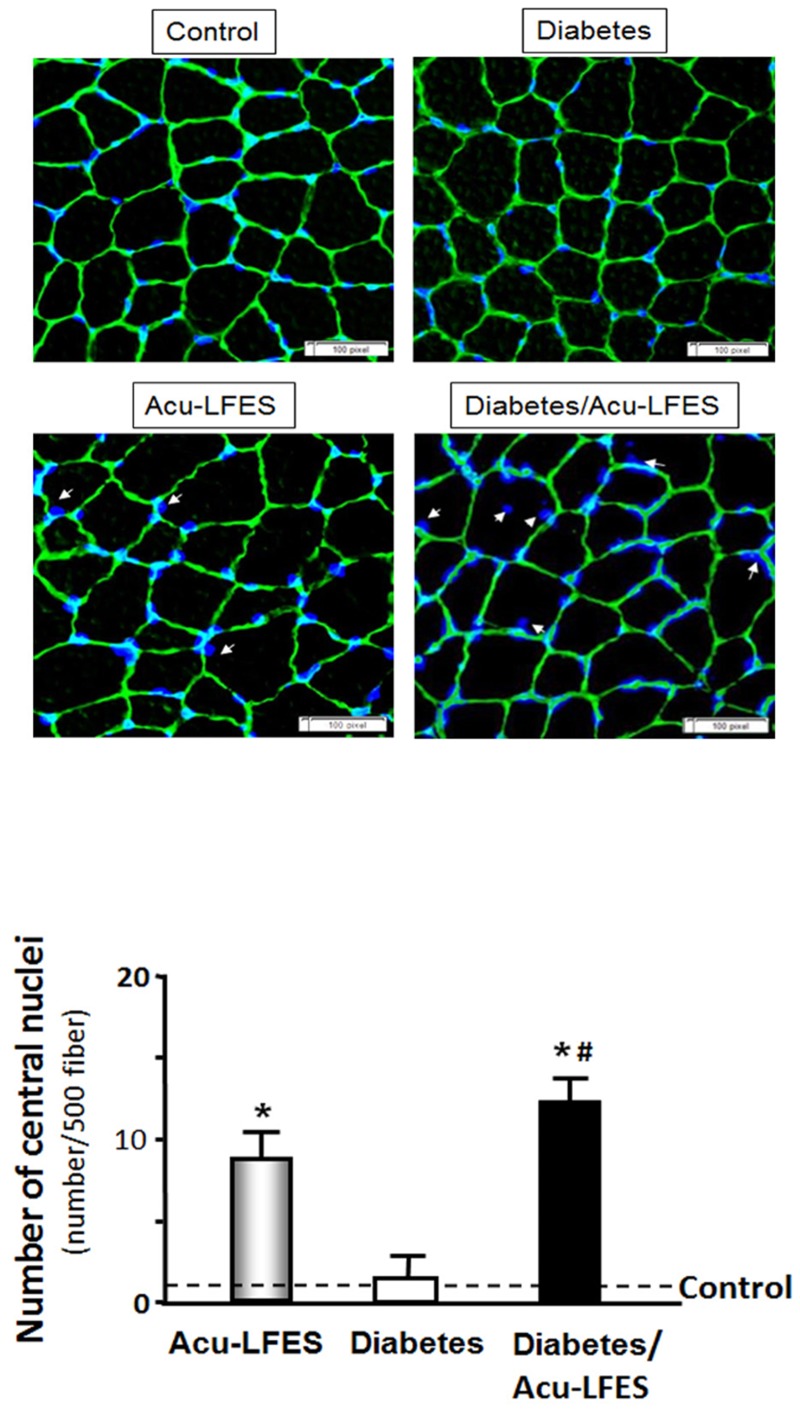
Acu-LFES increase satellite cells migration in normal and diabetic mice. A representative cross-sections from EDL muscles after staining for laminin (green) and counterstaining with DAPI (blue) are shown in control, Acu-LFES, diabetes or diabetes/Acu-LFES mice. The white arrows point to central nuclei inside of myofibers which indicates satellite cells migration. The bar graph shows the nuclei number inside (central nuclei) per 500 muscle fibers (Bars: mean ± s.e.; n = 9; # = p<0.05 vs. control).

Myofibers containing central nuclei indicate muscle regeneration. Under normal control conditions, satellite cells, also known as muscle stem cells, are generally located at the periphery of the myofibers. Upon initiation of myogenesis by Acu-LFES, satellite cells migrate into the myofibers (Fig 4). We found that central nuclei are apparent in muscles of both diabetic and non-diabetic mice treated with Acu-LEFS. However, the percentage increase in central nuclei was higher in the muscles of diabetic/Acu-LFES than of non-diabetic/Acu-LFES mice. These results indicate that Acu-LFES prevents muscle atrophy partly by stimulating myogenesis.

### Acu-LFES upregulates protein synthesis-related proteins in diabetic mice

**Fig 5:**
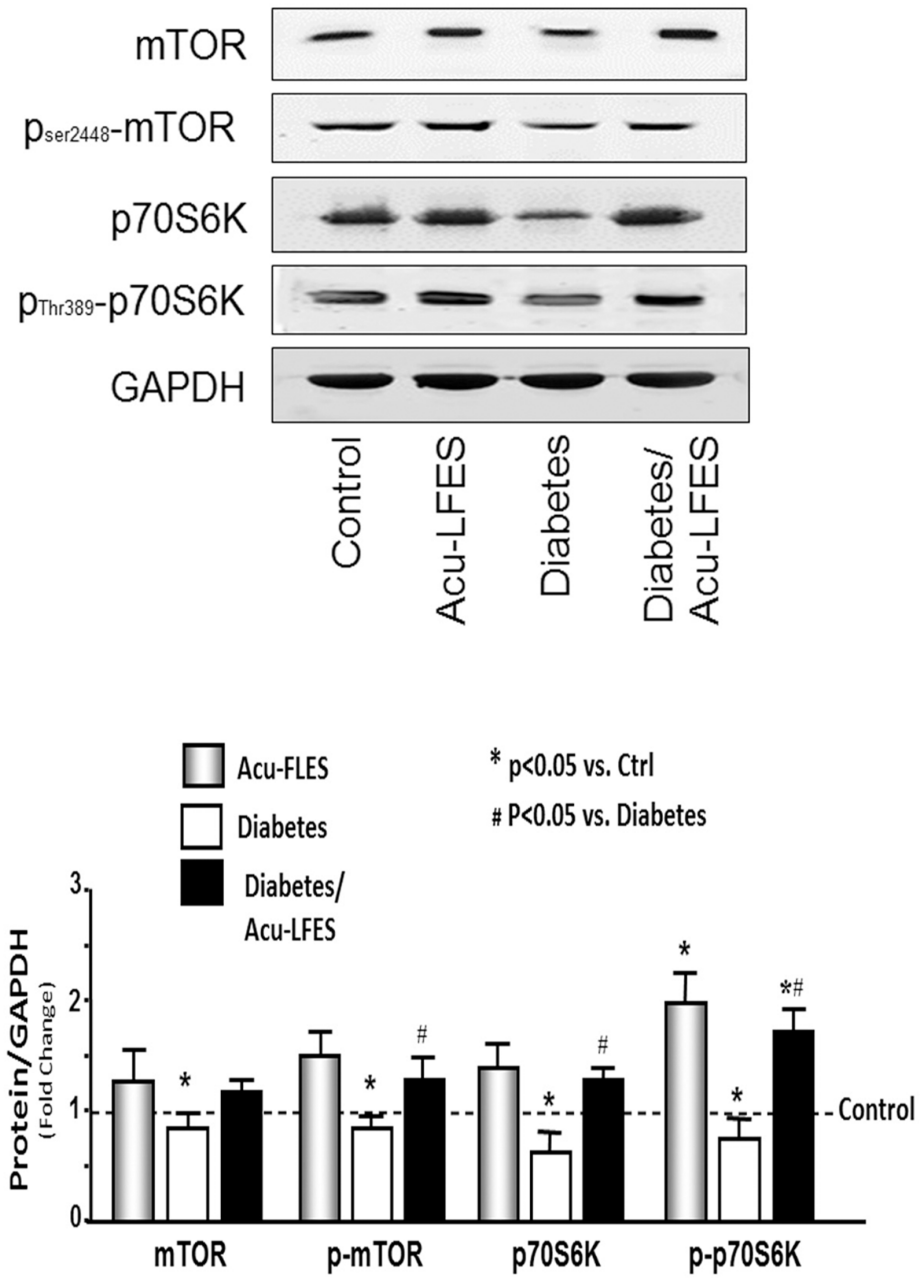
Acu-LFES improves protein synthesis markers in skeletal muscle of diabetic mice. The protein synthesis related markers mTOR, p-mTOR, p70S6K and p-p70S6K were measured by western blotting in combined gastrocnemius and EDL muscle lysates from control, Acu-LFES, diabetes or diabetes/Acu-LFES mice. The bar graph compares the densities of protein bands in each group expressed as a fold-change from levels in control mice which is represented by a line at 1-fold. All band densities were normalized to the density of GAPDH (Bars: mean ± s.e.; n = 12/group; * = p<0.05 vs. control and # = p<0.05 vs. diabetes).

Building muscle mass requires an increase in protein synthesis as well as the activation of myogenesis signaling. We tested protein synthesis-related proteins in all groups of mice (Fig 5). Both the phosphorylation of the mammalian target of rapamycin (p-mTOR) and the phosphorylation of the p70S6 kinase (p-p70S6) were significantly decreased in diabetic mice. The p-mTOR was increased 1.5-fold in muscles of non-diabetic mice treated with Acu-LFES over those of control mice; it was increased 1.6-fold in muscles of diabetic mice treated with Acu-LFES over those of diabetic mice that were not treated with Acu-LFES. The p-p70S6, which is downstream from mTOR in the protein synthesis pathway, was increased 2.0-fold in Acu-LFES—treated non-diabetic mice and 2.3-fold in Acu-LFES—treated diabetic mice over that of diabetic mice without Acu-LFES treatment. These results demonstrate that Acu-LFES counteracts the diabetes-induced decrease in the phosphorylation of mTOR or p70S6, thereby potentially increasing protein synthesis.

### Acu-LFES increases Akt phosphorylation in diabetic mice

**Fig 6:**
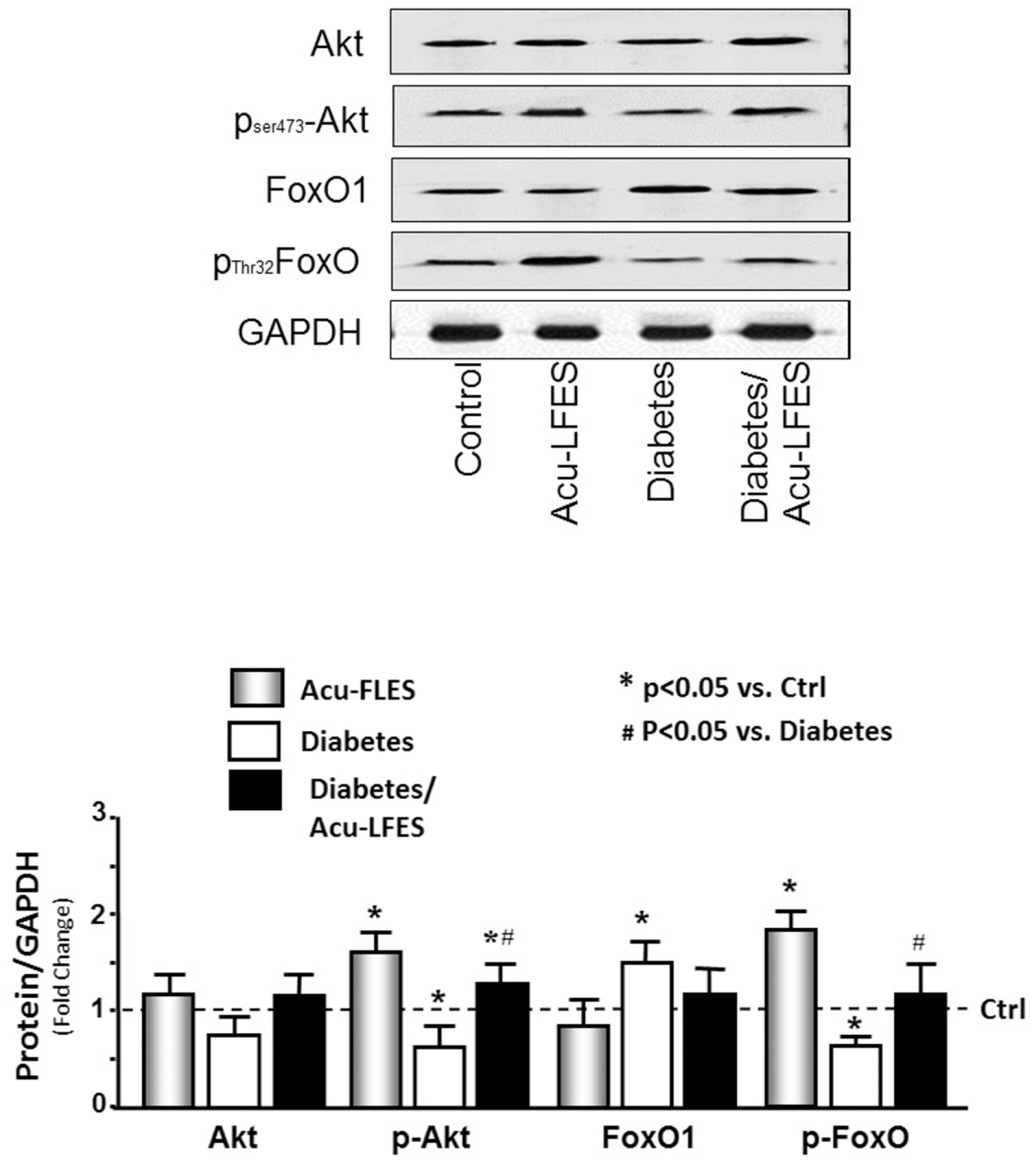
Acu-LFES improve Akt phosphorylation and inhibit FoxO activation in the muscle of diabetic mice. The protein metabolism related proteins Akt, p-Akt, FoxO1 and p-FoxO1 were measured by western blotting in combined gastrocnemius and EDL muscle lysates from control, Acu-LFES, diabetes or diabetes/Acu-LFES mice. The bar graph compares the densities of protein bands in each group expressed as a fold-change from levels in control mice which is represented by a line at 1-fold. All band densities were normalized to the density of GAPDH (Bars: mean ± s.e.; n = 12/group; * = p<0.05 vs. control and # = p<0.05 vs. diabetes).

Phosphorylation of Akt is important for muscle growth. Phosphorylation of FoxO is critical for prohibiting pro-catabolic functions of FoxO [18]. We assayed phosphorylation of these two proteins in all groups of mice (Fig 6). The phosphorylation of Akt was increased 1.6-fold in control muscle and 2.0-fold in diabetic muscle by Acu-LFES treatment over those of diabetic mice that were not treated with Acu-LFES. The Thr32 phosphorylation of FoxO1 was increased 1.9-fold in Acu-LFES—treated non-diabetic mice and 1.8-fold in Acu-LFES—treated diabetic mice over that of diabetic mice not treated with Acu-LFES. These results demonstrate that Acu-LFES improves protein synthesis by increasing AKT and FoxO1 phosphorylation.

### Acu-LFES upregulates the IGF-1 signaling pathway in skeletal muscle of normal and diabetic mice

**Fig 7:**
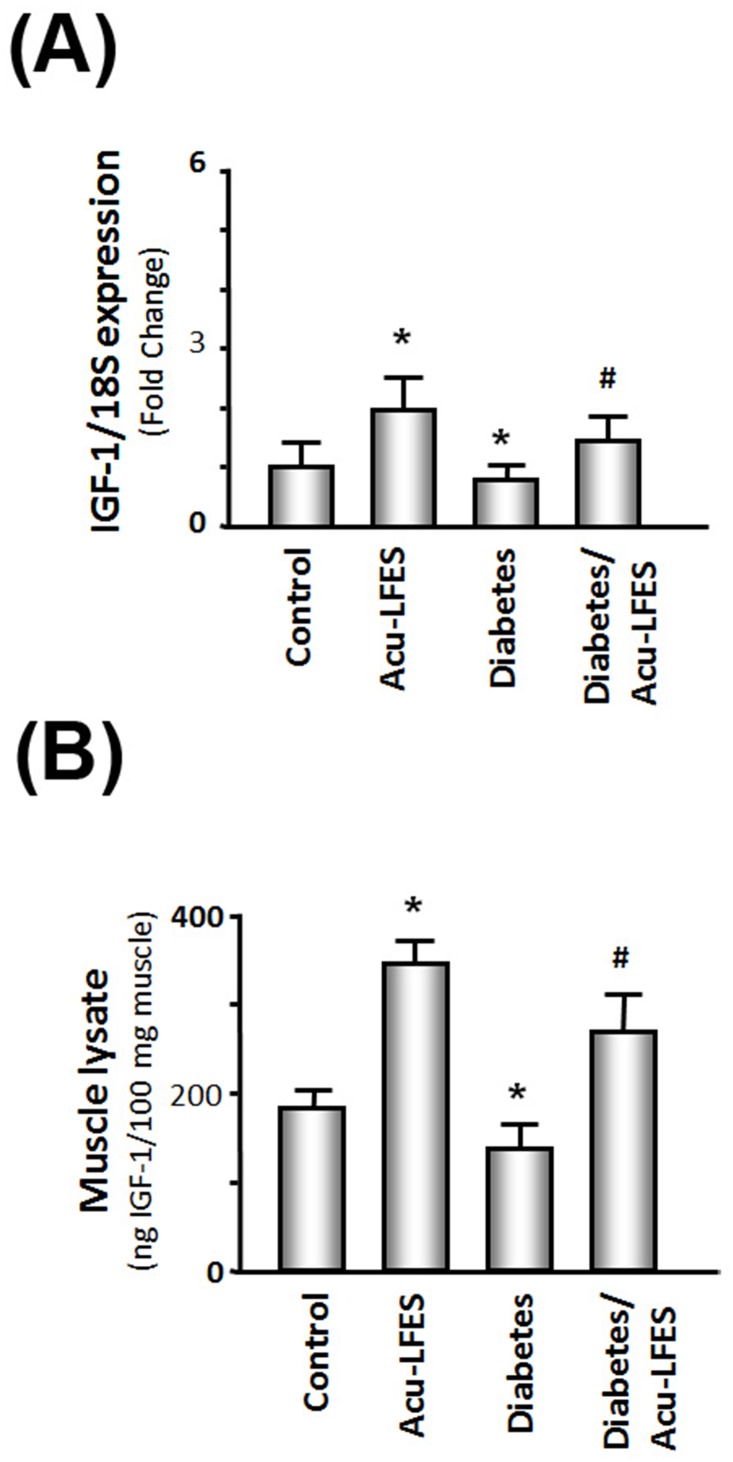
Acu-LFES upregulates IGF-1 mRNA and protein in the muscle of diabetic mice. Panel A: Total RNA isolated from combined gastrocnemius and EDL muscles of control, Acu-LFES, diabetes or diabetes/Acu-LFES mice were assayed for IGF-1 expression by real time qPCR. The bar graph shows mRNA from the muscles of each group of mice. Results are normalized to 18S RNA (Bars: mean ± s.e.; n = 9/group; * = p<0.05 vs. control and # = p<0.05 vs. diabetes). Panel B: IGF-1 protein levels were measured by ELISA in in combined gastrocnemius and EDL lysates from control, Acu-LFES treated normal controls (Acu-LFES), diabetes or diabetes/Acu-LFES mice. The bar graph shows the IGF-1 protein levels in each group. IGF-1 levels in the muscle lysates were normalized to the total protein concentration (Bars: mean ± s.e.; n = 9; * = p<0.05 vs. control and # = p<0.05 vs. diabetes).

To investigate how Acu-LFES increases muscle mass and activates the muscle regeneration process, we identified the mRNA expression of *IGF-1* using quantitative real-time PCR (qPCR). Acu-LFES increased *IGF-1* expression 2.0-fold in non-diabetic mice. *IGF-1* expression was 16% lower in diabetic mice than in non-diabetic mice. However, Acu-LFES reversed this trend; IGF-1 expression was increased 1.8-fold in diabetic mice treated with Acu-LFES over that of diabetic mice not treated with Acu-LFES (Fig 7). Protein levels of IGF-1 were measured by enzyme-linked immunosorbent assay in muscle lysates. IGF levels were 20% lower in diabetic mice than in control mice. Acu-LFES increased IGF-1 protein 1.9-fold in the muscles of diabetic mice indicating that IGF-1 is increased by Acu-LFES treatment in mice.

### Acu-LFES enhances the expression of microRNAs (myomiRs)

**Fig 8:**
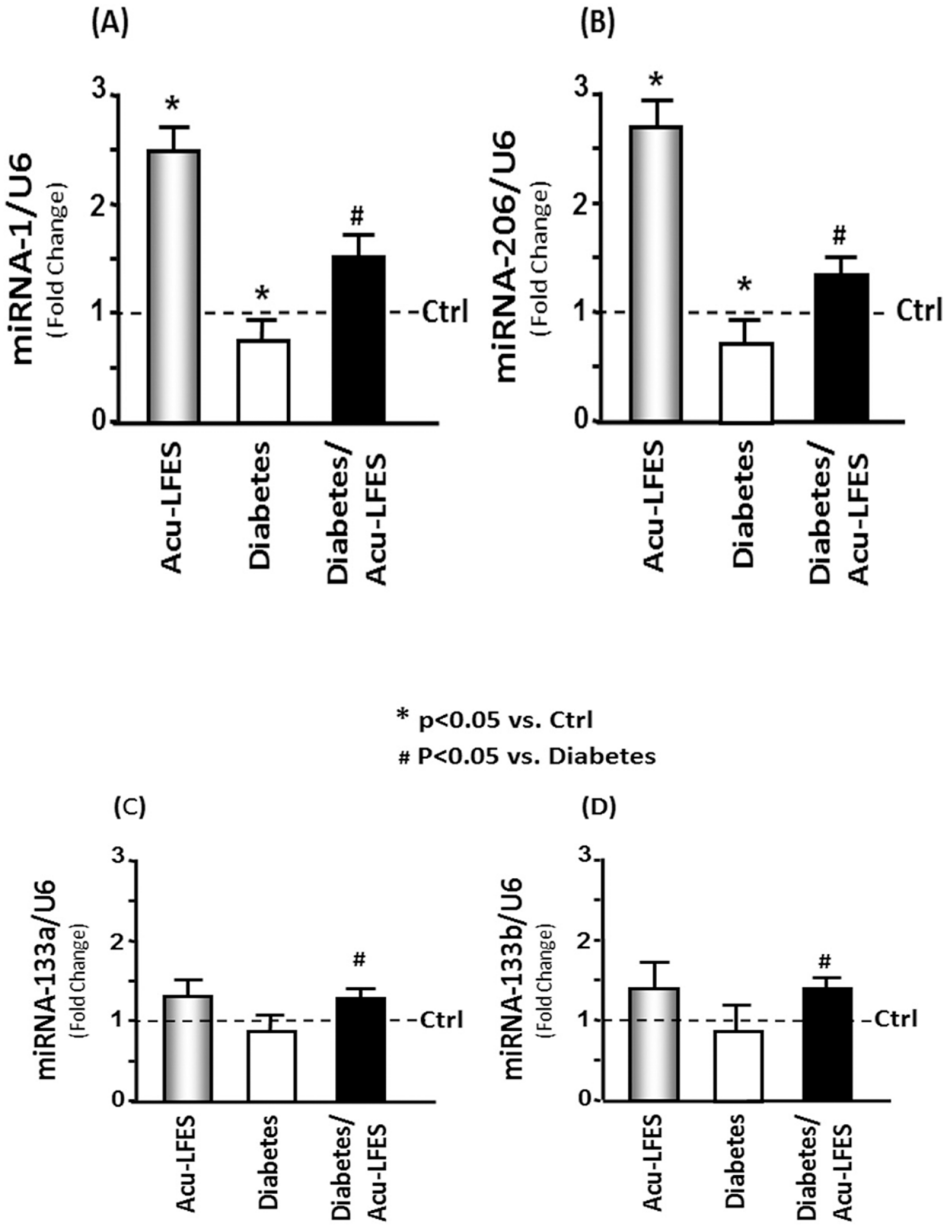
Acu-LFES increase miR-1 and miR-206 microRNA in the muscle of control and diabetic mice. Total RNA was isolated from combined gastrocnemius and EDL muscles of control, Acu-LFES, diabetes or diabetes/Acu-LFES, and then assayed for specific microRNA expression. miR-1 (A), miR-206 (B), miR-133a (C) and miR-133b (D) expressions were measured using real time qPCR with LNA-enhanced oligonucleotide primers. The bar graph shows microRNA levels in each group expressed as a fold-change from levels in control mice which is represented by a line at 1-fold. All band densities were normalized to the density of U6 RNA (Bars: mean ± s.e.; n = 6/group; * = p<0.05 vs. control and # = p<0.05 vs. diabetes).

Muscle regeneration is also regulated by myomiRs. To determine whether Acu-LFES stimulates the expression of muscle-specific microRNAs, we measured microRNAs by qPCR in muscle after 14 days of Acu-LFES (Fig 8). The expressions of miR1 and miR206 were significantly decreased in diabetic mice but were increased by Acu-LFES in non-diabetic mice (2.5-fold for miR1 and 2.7 fold for miR206) and diabetic mice (2.0-fold for miR1 and 1.8-fold for miR206). The expressions of both miR133a and miR133b in control mice were increased 1.3-fold by Acu-LFES, but the change was not statistically significant. However, Acu-LFES significantly increased miR133a and b expression in diabetic mice (1.5-fold for miR133a and 1.7-fold for miR133b). These data indicate that one of the methods by which Acu-LFES promotes muscle regeneration is the stimulation of myomiR expression.

## Discussion

The mechanisms that cause muscle protein loss in catabolic disease have been widely studied. Identification of common pathways of muscle wasting should lead to therapeutic strategies that are beneficial for all catabolic conditions. Other researchers and we have found that decrease of IGF-1 is a common pathway for muscle wasting, and upregulation of the IGF-1 signaling pathway counteracts muscle atrophy in diabetes and chronic renal failure in animal models [4, 5, 9, 19, 20]. In this study, we identified a simple non-pharmacologic treatment, Acu-LFES, which is able to increase IGF-1 signaling and prevent diabetes-induced muscle atrophy by stimulating muscle regeneration.

Much effort has been made to identify new therapeutic approaches to preventing muscle wasting. Pharmacologic treatments for muscle atrophy that have been studied in animal models and clinical trials include an anti-myostatin peptibody [21]; Stat3 inhibitor [22]; anabolic androgenic steroids such as nandrolone decanoate, a synthetic derivative of testosterone [23]; IGF-1 and its analogues [24]; an angiotensin-II receptor antagonist, losartan [25]; proteasome inhibitors [26, 27]; *MURF1* inhibitor PO13222 [28]; a caspase inhibitor called X-linked inhibitor of apoptosis protein [3, 10]; and antisense oligonucleotides [29]. However, pharmacologic treatments have adverse effects, and no simple and efficient treatments are currently in clinical use. Non-pharmacologic therapy, such as exercise, upregulates the IGF-1 signaling pathway and decreases myostatin in muscle tissue of animals [5, 30] and humans [31, 32], thus preventing muscle atrophy. Unfortunately, patients with severe disease are usually unable to exercise consistently. Therapies that can safely suppress muscle wasting in patients who are bedridden or in wheelchairs are needed.

Therapeutic acupuncture is widely used in the United States and around the world [33]. According to the National Institutes of Health Consensus Development Panel on acupuncture, the evidence of acupuncture’s value is sufficient to expand its use into conventional medicine [33]. In a British Acupuncture Council survey, no serious adverse events were reported after 34,407 acupuncture treatments, proving that acupuncture is a safe non-pharmacologic treatment [34, 35]. Acupuncture and Acu-LFES can decrease skeletal muscle atrophy induced by hind limb suspension in mice [36]. Electrical acupuncture treatment has been shown to suppress myostatin expression, which leads to satellite cell proliferation and skeletal muscle repair [37]. Previously, we performed Acu-LFES on mice with muscle atrophy induced by chronic kidney disease and found that Acu-LFES treatment improves muscle weight and function [14].

In the current study, we determined that Acu-LFES slows muscle wasting in diabetic mice by promoting muscle regeneration. Skeletal muscle is a postmitotic tissue. Mature muscles are composed of muscle fibers surrounded by a basal lamina that covers myofibrils and muscle precursor cells (satellite cells) [20, 38]. Satellite cells underneath the lamina are generally quiescent. However, in response to muscle injury or changes in growth factors (e.g., IGF-1), they begin to express myogenic regulatory factors (*MYOD*, *Myf5*, myogenin, and *MRF4*), leading to satellite cell proliferation, differentiation, and fusion to form myofibrils. In this way, myofibrils are repaired or enlarged. MYOD is one of the earliest markers of myogenic commitment and is also expressed in adult muscle in a muscle-specific manner. The influence of catabolic disease on the function of satellite cells has received limited attention. In a previous study, we provided evidence that satellite cell function is impaired by catabolic disease [20]. In the current study, we found that Acu-LFES promotes muscle regeneration and increases PAX7, which activates satellite cells and increases MYOD, myogenin, and eMyHC. This could promote satellite cell proliferation and differentiation.

The beneficial effects of Acu-LFES on muscle regeneration may be achieved by two mechanisms: upregulating IGF-1 or increasing muscle-specific microRNA (myomiR).

In this study, the gastrocnemius and EDL muscles were used together in the Western blot and PCR analyses. The gastrocnemius muscle was chosen because it is the major muscle in the distal hind limb. The EDL muscle was chosen because the two acupuncture points (positive: Yang Ling Quan, GB34; negative: Zu San Li, ST36) are very close to the EDL. The muscles were analyzed in a combined sample because this more accurately reflects the comprehensive muscle response to acupuncture treatment. We believe that LFES produces a nerve-borne effect that affects both muscles together. We found that Acu-LFES improves muscle regeneration by reversing the diabetes-induced suppression of IGF-1, p-Akt (protein anabolic markers), and p-mTOR (protein synthesis markers). Our results and those of other investigators have shown that IGF-1 plays a central role in controlling the muscle wasting of diabetes and other catabolic disease [4, 9, 18]. In general, an increase in IGF-1 signaling will counteract muscle atrophy induced by muscle injury and disease [4, 5, 9, 19, 20]. In the current study, we found that Acu-LFES upregulates IGF-1 mRNA, and protein. An increase in IGF-1 inhibits activation of the protein catabolic marker FOXO by increasing FOXO phosphorylation, thus providing a means of increasing muscle mass and function. An increase in IGF-1 will initiate muscle regeneration and protein synthesis and lead to increased muscle mass and function.

Acu-LFES appears to have the greatest effect on pre-existing muscle atrophy. Our results showed that muscle size is not induced in control with Acu-LFES—treated mice, despite the increases in IGF-1 and genes associated with regeneration proteins. The number of central nuclei was significantly higher in diabetic mice treated with Acu-LFES than in non-diabetic mice treated with Acu-LFES (Fig 4). Further study is required to explain this difference.

Several studies have revealed that microRNA is involved in the regulation of muscle regeneration [39]. MicroRNAs that are expressed mainly, but not exclusively, in muscle and that have significant impact on myogenesis are called myomiRs [39]. MyomiRs include miR1, -133, -206, -208, and -499. In a rat skeletal muscle injury model, injection of double-stranded miR1, miR133, and miR206 into muscle induced MYOD, PAX7, and myogenin, leading to increased muscle regeneration [40]. One study provided evidence that miR1 and miR206 directly target PAX3, leading to initiation of the myogenic program [41]. A recent study demonstrated that miR1 and miR206 play a major role in myoblast differentiation by regulating multiple target genes [42]. Evidence that miR206 promotes skeletal muscle regeneration is found both in mice with normal muscles and in those with Duchenne muscular dystrophy [43]. In the current study, we found that miR1 and -206 were decreased in the muscle tissue of diabetic mice. Acu-LFES not only increased miR1 and -206 in muscles of normal mice but also prevented the decrease of myomiRs in muscles of diabetic mice.

The benefits of acupuncture to diabetic animals and humans may extend beyond increasing muscle regeneration. In a randomized, controlled clinical trial, Man et al. found that electro-acupuncture improves insulin sensitivity [44]. Their study used electric acupuncture at bilateral ST36 and SP6 acupoints in 52 female patients and found that acupuncture was associated with prevention of hyperglycemia because of increased insulin sensitivity. The improvement of insulin sensitivity has been confirmed by other studies [45].

As with any treatment, acupuncture should be used with proper precautions. In the course of our studies, we have found that acupuncture has advantages and disadvantages. This may be the reason that previous acupuncture studies have sometimes shown equivocal results [46]. We found that Acu-LFES can activate macrophages and cause an acute pro-inflammatory response [14]. This pro-inflammatory response could promote myogenesis; however, long-term inflammation could also cause muscle damage. The timing and intensity of Acu-LFES seem to be strongly associated with the degree of inflammatory response. The long-term benefits or adverse effects of Acu-LFES were not established in this study.

Our study has some limitations. First, this study does not distinguish between the effects of acupuncture and electrical stimulation. It is possible that acupuncture actually induces damage and that LFES induces the increase in muscle size. Second, the effect on muscle mass of upregulation of miR1 and miR206 by Acu-LFES should be studied further. MyomiRs may increase signal differentiation during embryogenesis, but other studies have shown that overexpression of miR1 and miR206 inhibits IGF-1 expression [47]. In the current study, we found that IGF-1 signaling actually increased.

In conclusion, Acu-LFES ameliorated diabetes-induced skeletal muscle atrophy by improving muscle regeneration, leading to increased muscle mass and function. The increased muscle regeneration capacity is due to upregulation of the IGF-1 signaling pathway and an increase in the expression of myomiRs.
